# A global dataset of seaweed net primary productivity

**DOI:** 10.1038/s41597-022-01554-5

**Published:** 2022-08-06

**Authors:** Albert Pessarrodona, Karen Filbee-Dexter, Kira A. Krumhansl, Morten F. Pedersen, Pippa J. Moore, Thomas Wernberg

**Affiliations:** 1 UWA Oceans Institute and School of Biological Sciences, University of Western Australia, Crawley, Western Australia 6009 Australia; 2 Institute of Marine Research, His, Bergen, Norway; 3 Fisheries and Oceans Canada, Bedford Institute of Oceanography, Dartmouth, Nova Scotia Canada; 4 Department of Science and Environment, Roskilde University, Universitetsvej 1, DK-4000 Roskilde, Denmark; 5 School of Natural and Environmental Sciences, Newcastle University, Newcastle-Upon-Tyne, NE1 7RU UK

**Keywords:** Community ecology, Carbon cycle

## Abstract

Net primary productivity (NPP) plays a pivotal role in the global carbon balance but estimating the NPP of underwater habitats remains a challenging task. Seaweeds (marine macroalgae) form the largest and most productive underwater vegetated habitat on Earth. Yet, little is known about the distribution of their NPP at large spatial scales, despite more than 70 years of local-scale studies being scattered throughout the literature. We present a global dataset containing NPP records for 246 seaweed taxa at 429 individual sites distributed on all continents from the intertidal to 55 m depth. All records are standardized to annual aerial carbon production (g C m^−2^ yr^−1^) and are accompanied by detailed taxonomic and methodological information. The dataset presented here provides a basis for local, regional and global comparative studies of the NPP of underwater vegetation and is pivotal for achieving a better understanding of the role seaweeds play in the global coastal carbon cycle.

## Abstract (summary)

**Table:** 

Measurement(s)	Seaweed net primary productivity
Technology Type(s)	photorespirometry, biomass accumulation, eddy covariance
Factor Type(s)	Annual per-area net primary productivity
Sample Characteristic - Organism	Phaeophyceae • Rhodophyta • Chlorophyta
Sample Characteristic - Environment	marine biome
Sample Characteristic - Location	Global

## Background & Summary

NPP is a major driver of ecological functioning and a key flux in the global carbon cycle^1^. The advent of remote sensing technologies has facilitated the measurement of terrestrial^2–4^, freshwater^5,6^, and oceanic^7,8^ NPP at unprecedented scales, with most global models of NPP available to date relying on space-based observations^4,6^. In contrast, the magnitude, patterns and determinants of spatial and temporal variation of primary productivity in the coastal ocean remains poorly understood^9^. This is particularly true for submerged vegetated habitats such as seaweed forests or seagrass beds, which are important contributors to coastal productivity globally^10^, but whose NPP cannot be measured accurately by satellite sensors as these perform poorly at shallow depths where submerged vegetation occurs (0–30 m)^11^. Rather, most observations rely on *in situ* measurements^12^. Existing measurements of coastal vegetation NPP vary however in methodology and are usually reported in different units, hindering our understanding of the role these habitats play in the carbon cycle and how it compares to other primary producers^13^. Additionally, the majority of measurements are conducted at local scales, which means compilation of multiple local-scale datasets is required to unravel larger spatiotemporal patterns^12^.

Seaweeds form the largest and most productive underwater vegetated habitat on Earth, drawing a flux of CO_2_ comparable to the Amazon rainforest every year^14^. The carbon assimilated through this production fuels local marine food webs^15,16^ and can constitute a trophic subsidy to areas with low primary production such as soft-bottom communities^17^. Recent studies also suggest that seaweed carbon makes important contributions to oceanic carbon export^18^, with some estimates identifying seaweeds as major contributors to oceanic carbon sequestration^19^. This has reopened the debate on their potential use as carbon dioxide removal and/or climate change mitigation tools^20,21^, although great uncertainties exist in the carbon fluxes they underpin^19^. Indeed, despite the fact that it has been more than 70 years since seaweeds were shown to be amongst Earth’s most productive organisms^22–24^, we still know little about how their NPP varies across taxa, space and time^25^. Previous attempts to collate seaweed NPP data at large spatial scales have been geographically restricted (e.g. refs. ^13,26^) or focused on specific taxa (e.g. refs. ^27,28^). These limitations have precluded a global understanding of the patterns and determinants of NPP across seaweed taxa, which is in urgent need to inform on the promising potential of seaweeds.

Here we describe the most comprehensive global dataset of marine macroalgae NPP gathered to date. Data was obtained from the primary literature or provided directly by authors and contains records from a total of 246 taxa from 429 sites in 72 different ecoregions. Measurements of seaweed NPP were collected at the taxa level and reflect per-area productivity rates across a range of depths and seaweed groups. Each record is accompanied by detailed descriptions of the methodology used and is classified into habitat groups depending on the growing substrate, vegetation height and dominant vegetation at the study site. The dataset can be used to answer a range of long-standing questions, from investigating productivity patterns across taxa, methods, locations, and habitats, to building the first global NPP products for shallow submerged vegetation^29^. Additionally, as nearly all records have geographic coordinates, NPP measurements can be linked to available environmental data layers (e.g. ref. ^30^).

## Methods

### Data compilation

An extensive search of published reports, PhD thesis, and the peer-reviewed literature was performed to capture studies dealing with the net primary productivity or biomass production of wild (i.e. not cultured) marine macroalgae. First, a formal search was performed in the Scopus database using the search terms “primary AND product* OR growth or npp AND (seaweed OR alga* OR kelp OR rocky AND reef OR turf OR temperate AND reef OR coral OR polar OR Arctic)”, which yielded 498 entries (April 2022). We then filtered the query by searching for relevant content in the title and abstract, yielding a total of 69 studies. Further searches were conducted in the China National Knowledge Infrastructure database (CNKI), J-STAGE repository (Japan), and Scientific Electronic Library Online (SciELO) to capture studies with English abstracts from underrepresented regions such as Asia and South America. Additional studies were included from existing reviews on the productivity of tropical^31,32^, temperate^13,33^ and polar seaweeds^34^ and from being cited in the scanned papers. Finally, we included a few more studies from MSc or PhD thesis, the authors’ unpublished data, and other published reports based on our knowledge of the research field.

### Data selection and quality control

**Fig. 1:**
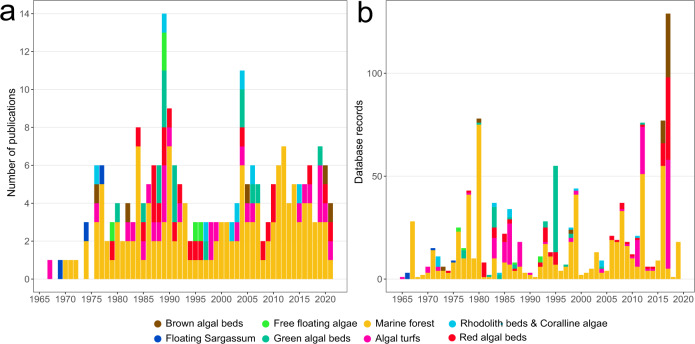
Temporal coverage of seaweed NPP measurements conducted at different habitat types and by tidal level (intertidal or subtidal), which are indicated in different colours. (**a**) Number of database records (i.e. a measurement of NPP per taxa, depth, site, year and method) depending on when the measurements were conducted. (**b**) Number of studies by date of publication (note not all data came from published studies).

Given that our analysis was centered on patterns of annual areal carbon production by seaweeds, each of the potentially relevant studies was then evaluated against the following set of criteria to determine if they could be included in the final dataset. First, studies had to examine seaweed NPP or biomass accumulation on a per area basis. This criterion excluded studies examining biomass-specific productivity rates (e.g. refs. ^26,35^) unless those rates were applied to standing biomasses or covers in the field (e.g. ref. ^36^). Second, studies had to provide discrete estimates of NPP at the primary producer level (i.e. seaweed species or assemblage) with minimal interference of other photosynthetic or heterotrophic organisms. This criterion excluded studies examining net ecosystem primary production (NEP) and metabolism when the NPP of the seaweed component could not be accurately determined. Such studies usually relied on diel dissolved oxygen measurements in the water column (e.g. refs. ^37,38^), which often cannot resolve which organisms are responsible for primary production (but see ref. ^39^). An exception were oxygen measurements conducted directly above seaweed-dominated benthos (e.g. Aquatic Eddy Covariance method) with little heterotrophic respiration^40,41^. Third, studies had to capture seasonal variability in NPP across the year. This criterion excluded studies conducted at a single point in time, month or season, with the exception of studies concerning annual species where the growth or biomass accumulation was measured at the end of their life-cycle (i.e. the maximum period of growth). Fourth, quantification of productivity had to be performed *in situ* or outdoor mesocosms mimicking natural conditions. This criterion excluded laboratory-only experiments, aquaculture yields, model estimates (e.g. Ecopath models) and field studies in which the natural environmental conditions were experimentally modified (e.g. nutrient enrichment, acidification, sediment additions). Fifth, details of the specific sampling location and measuring method had to be provided. Sixth, studies had to provide new data not previously reported in other publications. This criterion excluded reviews, meta-analysis, as well as studies approximating NPP based on rates obtained elsewhere. After applying the criteria above, our final filtered dataset featured 1,084 records from 237 independent studies published between 1967 and 2022 and covered a range of seaweed vegetation types (Fig. 1a,b).

Available data were extracted into an excel template from the suitable articles’ text, tables, figures (using the graph digitizing tool *Webplot Digitizer*^42^) or supplementary material. In our study, a record was considered to be the aerial net primary productivity of a taxon over the course of a year. If the data in a given study was not directly reported as annual rates, these where computed based on the monthly, bimonthly or seasonal means, with the corresponding standard deviation also being computed. The sampling effort (frequency of measurements throughout the year) was also recorded as it may have impacted the estimates’ accuracy. Data were entered into the template in the same units as the original source, but were also standardized to annual areal carbon production (i.e. g C m^−2^ y^−1^) to facilitate comparison. Values reported in fresh (16% or records) or dry (54% of records) weight (FW, DW, respectively) were converted to carbon units. Conversion factors provided in the studies were preferably used, but otherwise these were derived from the a single extensive database^43^ to minimize variability. Species or genus-specific factors were used in most cases, but family- and order-specific factors where occasionally used when these were not available for a given species. Metadata describing the depth; substrate; sampling year and season; taxonomy; study site and its geolocation; measuring method; and data extraction procedure were attached to each individual row. When a given value was not available, it was entered as “NA”. If a study reported NPP from multiple taxa, depths, sites, methods or time points, these were entered as separate case studies (separate rows). NPP of taxa within the same sample plot (e.g. multi-species *Sargassum* bed, kelp and understory algae) was also entered as separate records, but a specific column was created to denote that data would require summation of the rows to yield total areal productivity of that plot.

A site was defined as a single location where NPP was measured using the criteria above, with its geographic coordinates being added as metadata. If these were not directly provided in the article, we used the maps and/or description of the study locations to approximate their coordinates on Google Maps, noting also that these were approximations in the record’s metadata. NPP records across depths were considered to be within the same study site as long as measurements were within 30 m of each other. Each independent site was given a unique ID within each study.

As different sampling methods measure different aspects of photosynthesis and carbon assimilation^13^, we also recorded the method used to estimate each value of NPP. These were grouped into several subcategories, which mostly fell into two basic approaches: photorespirometry and biomass accumulation (Table 1). Photorespirometry-based methods measure direct carbon assimilation or oxygen evolution (which is later converted to carbon units based on the photosynthetic quotient), while biomass accumulation measures only the carbon destined to plant growth (mostly blade growth), and thus is expected to always yield lower estimates of NPP^44^. Biomass accumulation approximates well true NPP when carbon demand for growth matches carbon fixation^45^, but increasingly diverges when there is surplus of carbon derived from photosynthesis (e.g. in high light conditions) and carbon is directed towards other pathways (e.g. dissolved organic carbon exudation^46^). It is worth noting that both photorespirometry and biomass-accumulation-based methods are typically conducted at small spatial scales (1s m; plant or assemblage level), and therefore may not capture habitat-scale (10s m) NPP. Studies measuring oxygen and carbon fluxes directly over the water column or benthos (e.g. Aquatic Eddy Covariance) may provide better estimates of whole-ecosystem productivity, but these rarely resolve the taxon-specific contributions to productivity (but see ref. ^37^). An overview and discussion of the advantages disadvantages of each method is provided elsewhere (e.g. refs. ^13,47,48^).

**Table 1:** Summary of the methods to estimate seaweed NPP in our database.

General Method	Method	Description	Examples
Biomass accumulation	Single Harvest	Production is assumed to be equal to the maximum standing biomass after a period of time. Production can be estimated by outplanting tiles into the field and quantifying their algal biomass in a given timeframe, or by harvesting annual species when they reach the end of their life cycle	^53,54^
	Periodic Harvest	Periodic harvests of standing biomass over short time scales. Changes in standing biomass are attributed to growth or losses. Production can be estimated by subtracting the maximum and minimum biomass achieved, summing of all positive increments, or by counting individuals of a cohort and their mean weight through time (Allen method)	^55–57^
	Commercial harvest	Periodic harvests of standing biomass but targeting certain vegetative structures. Plants are not cultured but rather grown on the reef	^58,59^
	Tagging	Individual-plant increases in weight are followed through time by tagging, staining or punching holes in the plant. The mean individual increases in biomass are then multiplied by plant density to obtain areal rates	^60,61^
Photo-respirometry	Gas evolution (*in situ*)	Measurements of changes in dissolved oxygen (or more rarely CO_2_) of individuals or communities enclosed in transparent benthic chambers. Measures true NPP (carbon assimilation) by subtracting gross primary productivity from respiration. Respiration rates are obtained by enclosing individuals in dark chambers	^62,63^
	Gas evolution (mesocosm)	Measurements of changes in dissolved oxygen in individuals maintained in outdoor mesocosms with flow through seawater and field-like levels of irradiance	^64,65^
	Gas evolution (modelling)	Relationship between photosynthesis and irradiance established *in situ*, and photosynthesis modelled based on irradiance changes throughout the year	^66^
	Isotopes	Thalli are submerged in water enriched with isotopes and uptake by macroalgal tissue is measured after a given period of time. Measures true NPP as well as carbon isotope tracers (^14^C or more rarely ^13^C)	^67,68^
Aquatic Eddy Covariance	Aquatic Eddy Covariance	Measurements of changes in dissolved oxygen in directly over the benthos at high temporal resolution, integrating fluxes over large areas of the seafloor (10s m^2^)	^40,41^

Studies and taxa were also classified according to the habitat where measurements were performed using the information given within the published article (Table 2). Habitat categories were defined based on key structural parameters like vegetation height, the dominant vegetation (e.g. brown, red or green algae) as well as their position within the water column (benthic or pelagic). Within a study, taxa from different groups could be classed in the same habitat (e.g. canopy, epiphytes and understory algae all being part of a “marine forest”) unless they formed distinct patches within the habitat matrix (e.g. red algal bed patches interspersed with marine forests^49,50^), or the study examined different depth bands, sites or habitats. When incubations of different taxa were performed in isolation within a study, these were independently assigned a habitat category.

**Table 2:** Definitions for the habitat type category. Categories were based on vegetation height, dominant vegetation (brown, red or green algae) as well as their position in the water column (benthic or pelagic).

Habitat type	Description	Examples
Marine forest	Vegetation dominated by large canopies formed by brown algae from the orders Laminariales, Fucales, Tilopteridales and Desmarestiales. Includes understory and epiphytic taxa associated with the canopies.	Kelp & *Sargassum* forests
Brown algal beds	Low-lying vegetation dominated by brown algae	*Padina, Dictyota* beds
Red algal beds	Low-lying vegetation dominated by red algae	*Gelidium, Gracilaria* beds
Algal turfs	Low-lying vegetation dominated by aggregations of single or multiple species of short algae from different groups, forming a complex matrix	Algal turfs, epilithical algal matrix
Green algal beds	Vegetation dominated by attached green algae, including Halimeda biohermes	*Caulerpa* beds, *Halimeda* bioherme
Rhodolith beds & coralline algae	Habitats of coralline algae and rhodolith beds	Coralline barrens
Floating Sargassum	Pelagic Sargassum rafts (*S. fluitans*, *S. natans*)	*Sargassum* rafts
Other floating algae	Other free-floating aggregations of algae on the bottom or at the sea surface	Ulva blooms

## Data Records

The dataset, together with a reference list of all the studies included in it, is publicly accessible for download in the Figshare repository^51^.

### Taxonomic coverage

**Fig. 2:**
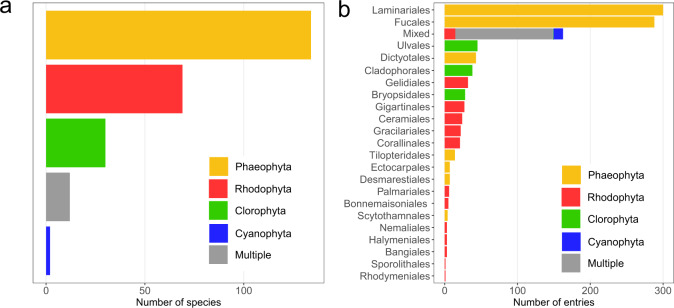
Taxonomic coverage of the database. Multiple denotes taxonomic groupings that involve species from different phyla (e.g. algal turfs).

The database contains NPP information for >240 species or taxonomic entities (e.g. crustose coralline algae, algal turf), from 49 families, 26 orders and all major seaweed groups and functional forms. The majority of species with NPP records are brown algae (55%; kingdom Phaeophyta) (Fig. 2a), with just over half the database being composed of records from the orders Laminariales and Fucales (558 records, Fig. 2b)

### Spatial and temporal coverage

**Fig. 3:**
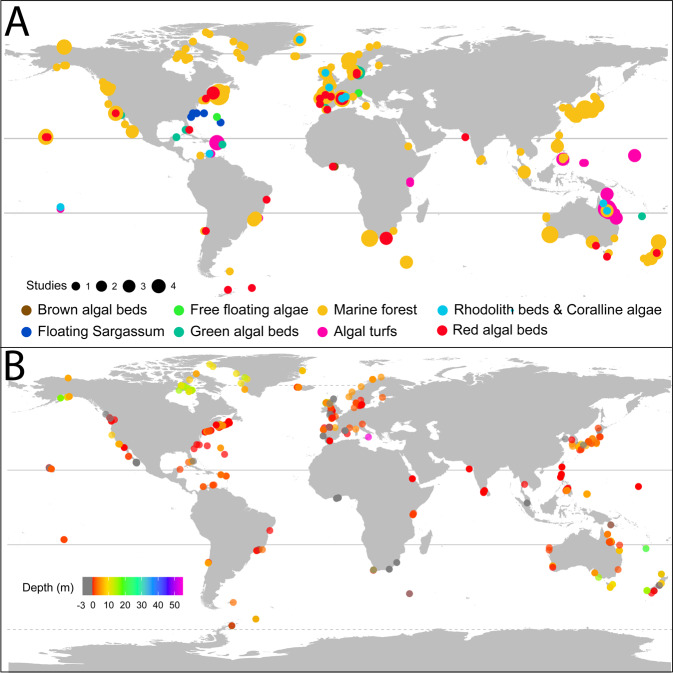
Location (**A**) and depth (**B**) of the study sites included in the database. Measurements conducted in the intertidal (i.e. above sea level are indicated in grey).

The dataset contains NPP data from 429 sampling sites (Fig. 3A) spanning from the high intertidal (3 m above mean sea level) down to 55 m (Fig. 3B). Sites span all major oceanographic realms and are distributed from the poles to the tropics, with most of the records concentrated in temperate latitudes 40–60° and concerning marine forests. The vast majority of studies measured NPP over 1-2 years. Only 2% of records report measurements conducted ≥3 years, and only three records report continuous NPP measurements >10 years. The temporal resolution of the measurements conducted within the sampling period varies from biweekly to annual measurements.

### Data collection sources and methods

**Fig. 4:**
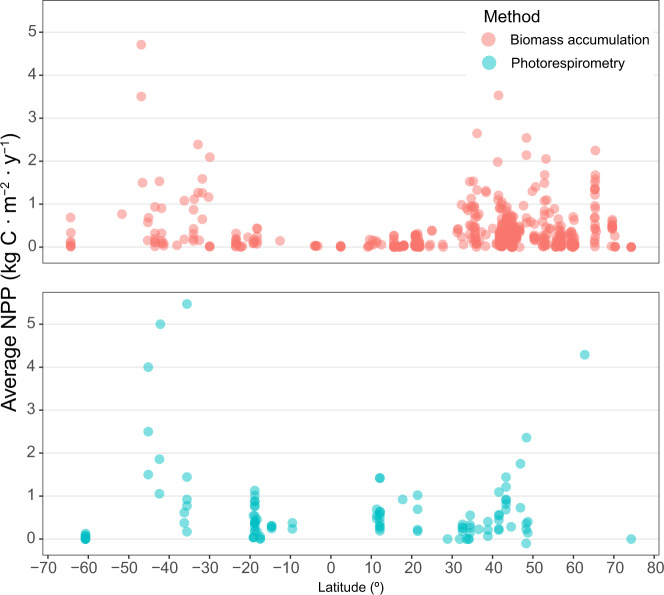
Latitudinal patterns of observed NPP depending on measuring methods. Dots indicate the average NPP of a study conducted within a given location.

**Fig. 5:**
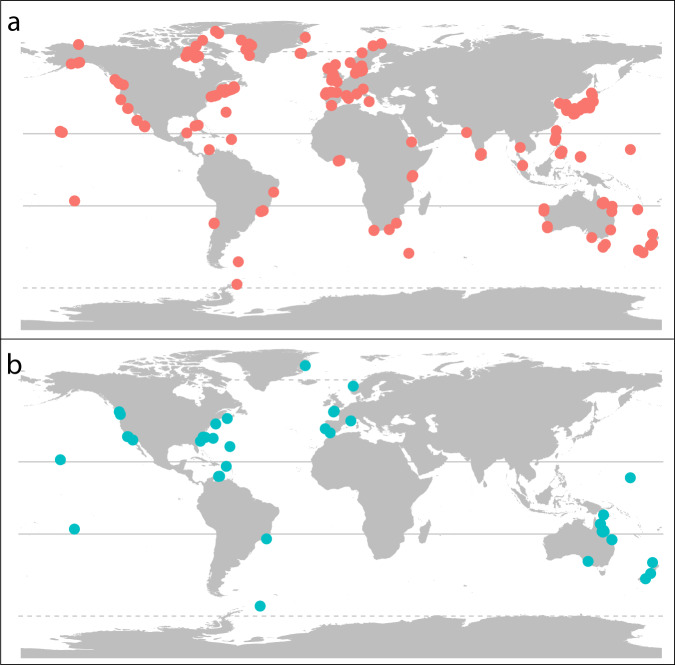
Distribution of observations depending on the methodology applied to measure NPP. (**a**) Biomass-accumulation-based and (**b**) Photorespirometry-based methods.

**Fig. 6:**
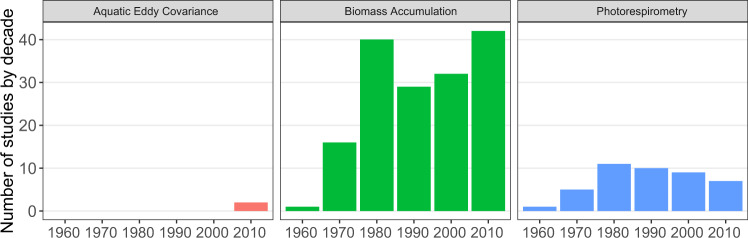
Number of studies measuring seaweed NPP per decade grouped by broad methodology.

Records were mostly extracted from the published literature (94%), followed by PhD and MSc thesis (2.5%), unpublished personal data (2.5%) and a minor fraction from published reports in the grey literature. Most of the data were sourced from tables and text (73% of records), whilst the rest was extracted from graphs (20%) or from raw data (7%). The vast majority of NPP records in the database were obtained using biomass-accumulation-based methods (87%), followed by photorespirometry-based methods (12.9%), with only a minor fraction of records using both methods and Aquatic Eddy Covariance (n = 2). While biomass accumulation and photorespirometry measure different aspects of carbon assimilation, NPP patterns from both methods are largely consistent across latitude (Fig. 4). Biomass accumulation measurements are well distributed globally (Fig. 5a), while photorespirometry-based measurements are common in coral reefs (mostly on algal turfs), the open ocean (pelagic* Sargassum* spp. rafts), and a few other temperate locations (Fig. 5b). Despite its limitations^48^, biomass accumulation remains the most widely used methodology to estimate NPP, with photorespirometry-based studies showing a small decline over time (Fig. 6). This may be due to the rise of more novel methods resolving gas fluxes in marine ecosystems at higher temporal and spatial resolution (e.g. Aquatic Eddy Covariance). Adoption of these relatively novel methods in seaweed habitats is still not widespread however^52^, possibly due to the their relatively high cost and current inability to be deployed in wave-highly-exposed environments where many seaweeds thrive.

## Technical Validation

The database was curated by the authors, with each of the records identifying who entered the data in the “Person_entering_data” column. We used templates to minimize spelling errors, inconsistencies, and incorrect values. Upon finalizing data entry, we conducted quality control by

i) Checking taxonomic names. The validity of taxa names was checked using the taxon match tool of the World Register of Marine Species (WoRMS) in May 2021. The names were corrected and updated if taxonomies had changed since publication of the study.
ii) Checking geographic coordinates. We projected the coordinates on a 1:10,000,000 shapefile of the world’s landmasses (EPSG:3857) checking they did not lay on land. When that was the case, we individually checked each value to ensure it was correct.
iii) Checking for duplicates. Records with identical NPP values for the same species and GPS coordinates were double checked for accuracy.
iv) Checking for outliers. Frequency histograms and quantile plots were generated to evaluate potential outliers. Records with very small (<1 g C m^−2^ y^−1^, i.e. 10% quartile) or large (<1,100 g C m^−2^ y^−1^, i.e. 95% quartile) NPP values were double checked for accuracy.

## Usage Notes

Each of the records (rows) in our database provides the average annual aerial NPP and standard deviation (when reported) of a given taxon at a given site, depth, year and study and by a given measuring method. Given that records were collected across multiple individual studies conducted at different time points, for certain purposes, some records may not be directly comparable. Thus, each record is also accompanied by a series of metadata describing the taxonomic information, geographic coordinates, description of the measuring method used as well as vegetation and substrate type. The dataset variables’ (columns) definitions and descriptions can be found in Table 3. When the taxa measured includes species from multiple genera, families, orders or classes, this is indicated as “Mixed”.

**Table 3:** Dataset variables and description.

Variable	Description
Vegetation_category	Habitat where the measurement was conducted. As per Table 2.
Substrate_category	The substrate on which seaweed grew. “Rock” if algae were found on rocky reefs, “Coral” if they occurred on coral reefs, “Floating” if they occurred as free-floating mats and “Sand” if they grew over sand or mudflats
Level	Intertidal or Subtidal. Subtidal is defined if Depth_min or Depth_max and is equal or smaller than zero metres below Chart Datum
Taxa	Species name as per WoRMS
Phyla	Phaeophyta, Rhodophyta, Chlorophyta or Multiple when including multiple types of phyla (e.g. algal turfs)
Order	Taxonomic order as per WoRMS
Family	Taxonomic family as per WoRMS
Genus	Taxonomic genera as per WoRMS
Multispecies	Refers to whether the study studied production of a single species, or an entire algal assemblage (e.g. algal turf, red algae). YES or NO.
Aggregation_required	Refers to whether the study provided the production by different species separately, but these required aggregation as they were part of the same area of seabed sampled (e.g. multi-species *Sargassum* bed, kelp and understory algae). YES or NO.
Site	Name of study site as described in the study
Site_ID_within_study	ID of a given site within a study reference
Latitude_decimal_degrees	Latitude converted to decimal degrees
Longitude_decimal_degrees	Longitude converted to decimal degrees
Original_coordinates	Whether the coordinates where provided in the study or were obtained by the authors via maps of the study areas referred by each study
Depth_min_m	Minimum depth in m. Negative values indicate above Chart Datum. If “NA”, depth not given in the study (we may know however if it was in the intertidal or subtidal)
Depth_max_m	Maximum depth in m. Negative values indicate above Chart Datum. If “NA”, depth not given in the study (we may know however if it was in the intertidal or subtidal)
Start_year	Year the first sample of the study was collected
End_year	Year the last sample was collected. Note that monthly samples collected over a year may still have the same starting and ending years.
Ann_sampling_freq:	Number of samples collected over a year time period. Ranges from 1 (annual sampling, e.g. for annual species) to 24 (i.e. biweekly samples)
Seasons	Seasons over which data was collected, corrected by hemisphere.
Data_mining_method	Method used to collect the data. “Graph digitizer”, if data was collected from figures in the manuscript, “Text”, if values were mentioned in the text of the manuscript, and “Raw data” if the authors were able to perform calculations based on the full dataset
Description	Description of how the study estimated NPP. It usually contains number of samples collected, and other experimental details (e.g. size of incubation bottles, types of plants selected…)
Reference	Abbreviated reference of the study
Production_method	Describes the method used to estimate NPP as per Table 1.
Prod_method_general	Biomass accumulation (BA) or Photorespirometry (PR)
Avg_NPP	Average NPP. Original production values given in the study, regardless of timeframe (hrs, days, months, year)
sted_NPP	Original standard deviation for the value given in the study, regardless of timeframe (hrs, days, months, year)
NPP_units	Original production values given in the study (e.g. mol O_2_ m^−2^ y^−1^, g C g^−1^ DW day^−1^, kg C m^−2^ y^−1^)
FW_DW_Conversion_factor	Conversion factor used to convert values into dry biomass. Whenever those where not provided in the study, we used the species —or, in a few cases, higher taxonomic denomination— mean ratios provided in ref. ^43^
DW_Carbon_Conversion_factor	Conversion factors used to convert values into carbon units. Whenever those where not provided in the study, we used the species — or, in a few cases, higher taxonomic denomination — mean ratios provided in ref. ^43^
Avg_NPP_kg_C_m2_y	Production values converted to kg C m^−2^ y^−1^
stdev_NPP_kg_C_m2_y	Production standard deviation converted to kg C m^−2^ y^−1^
Person_entering_data	Author that entered the data

Despite our efforts to obtain measurements across the globe, our dataset contains taxonomic, depth and geographical biases (Figs. 2, 3), with most records concerning brown algae from shallow depths (<10 m) and few records being available from South America, Africa, the Indian Ocean and Antarctica. We advise that researchers using the database should be aware of the influence these biases might have on their analyses.

## Data Availability

The code used to validate the dataset and make the figures in this manuscript is available at the Figshare repository^51^.
